# Intraspecific Polymorphisms of Cytogenetic Markers Mapped on Chromosomes of *Triticum polonicum* L.

**DOI:** 10.1371/journal.pone.0158883

**Published:** 2016-07-08

**Authors:** Michał Kwiatek, Maciej Majka, Joanna Majka, Jolanta Belter, Elżbieta Suchowilska, Urszula Wachowska, Marian Wiwart, Halina Wiśniewska

**Affiliations:** 1 Institute of Plant Genetics, Polish Academy of Sciences, Strzeszyńska 34, 60–479 Poznań, Poland; 2 Department of Plant Breeding and Seed Production, University of Warmia and Mazury in Olsztyn, Pl. Łódzki 3, 10–727 Olsztyn, Poland; 3 Department of Entomology, Phytopathology and Molecular Diagnostics, University of Warmia and Mazury in Olsztyn, Pl. Łódzki 3, 10–727 Olsztyn, Poland; Leibniz-Institute of Plant Genetics and Crop Plant Research (IPK), GERMANY

## Abstract

*Triticum* genus encloses several tetraploid species that are used as genetic stocks for expanding the genetic variability of wheat (*Triticum aestivum* L.). Although the *T*. *aestivum* (2n = 6x = 42, AABBDD) and *T*. *durum* (2n = 4x = 28, AABB) karyotypes were well examined by chromosome staining, Giemsa C-banding and FISH markers, other tetraploids are still poorly characterized. Here, we established and compared the fluorescence *in situ* hybridization (FISH) patterns on chromosomes of 20 accessions of *T*. *polonicum* species using different repetitive sequences from BAC library of wheat ‘Chinese Spring’. The chromosome patterns of Polish wheat were compared to tetraploid (2n = 4x = 28, AABB) *Triticum* species: *T*. *durum*, *T*. *diccocon* and *T*. *turanicum*, as well. A combination of pTa-86, pTa-535 and pTa-713 probes was the most informative among 6 DNA probes tested. Probe pTa-k374, which is similar to 28S rDNA sequence enabled to distinguish signal size and location differences, as well as rDNA loci elimination. Furthermore, pTa-465 and pTa-k566 probes are helpful for the detection of similar organized chromosomes. The polymorphisms of signals distribution were observed in 2A, 2B, 3B, 5B, 6A and 7B chromosomes. Telomeric region of the short arm of 6B chromosome was the most polymorphic. Our work is novel and contributes to the understanding of *T*. *polonicum* genome organization which is essential to develop successful advanced breeding strategies for wheat. Collection and characterization of this germplasm can contribute to the wheat biodiversity safeguard.

## Introduction

The importance of common wheat (*Triticum aestivum* L.) in food security and trade is crucial for many countries. Considering the progressive genetic erosion caused by narrow genetic diversity of this crop and the increasing number of races of wheat pathogens, there is a need to find new sources of variation. *T*. *aestivum* relatives have been used extensively to introduce adaptive genes into wheat mainly associated with biotic and abiotic stresses [1]. Bread wheat possess 42 chromosomes belonging to 3 genomes (2n = 6x = 42 chromosomes, AABBDD) and has no direct ancestor. In the accepted model for the allopolyploid speciation of hexaploid common wheat, the domesticated tetraploid emmer *T*. *dicoccum* or freerhreshing hard wheat *T*. *durum* migrated northeastward in association with the spread of agriculture across and beyond the Fertile Crescent region and came into contact with *Ae*. *tauschii* Coss (2n = 2x = 14, DD), which resulted in natural hybrid crosses [2, 3]. Tetraploid wheats evolved less than 0.5 million years ago through hybridization between *T*. *urartu* and *Aegilops speltoides* Tausch (2n = 2x = 14, SS). Those independent hybridization events are believed to be associated with the origin of *T*. *dicoccoides* (2n = 4x = 28, AABB) and *T*. *araraticum* (2n = 4x = 28, AAGG) [4]. The process of domestication resulted in originating several cultivated tetraploid wheats, as follows: *T*. *dicoccon* (Emmer wheat), *T*. *durum* (macaroni or hard wheat), *T*. *carthlicum* (Persian wheat), *T*. *polonicum* (Polish wheat) and *T*. *turanicum* (Khorasan wheat, Kamut®). Emmer wheat cultivation has declined today. It can be found only in some traditional farming communities mainly in Ethiopia, India and Yemen [5]. *T*. *durum* (macaroni or hard wheat) is a naked wheat, widely cultivated today for pasta production. *T*. *polonicum* (Polish wheat) is sporadically grown, and it is of marginal importance on the contemporary grain market. By contrast, *T*. *turanicum* (Kamut®) is mass produced mostly in organic farms in the United States, and its grain is used in the production of health foods [6]. Moreover, moderate or high levels of resistance against biotic stresses of *T*. *polonicum* and *T*. *turanicum* have been identified [7].

In recent times, ancient wheats such as: Emmer, Khorasan, Persian and Polish wheats and spelt captured the great deal of attention. These species are rediscovered by farmers because they grow under organic conditions, in marginal areas and sometimes get higher incomes with respect to modern wheat cultivars [8]. Moreover, the grain properties are perceived as more natural and healthy [9]. Furthermore, Emmer, Polish and Kamut® wheats could be an alternative source of Fusarium head blight (FHB) resistance [6]. FHB disease (caused mainly by *Fusarium graminearum* Schwabe; teleomorph *Gibberella zeae* (Schw.) Petch) is one of the most destructive fungal diseases of wheat worldwide. The disease affects both hexaploid bread wheat and tetraploid durum wheat and have caused serious losses in grain yield and quality [10, 11]. So far, reactions to FHB in these tetraploid wheats have been extensively evaluated only in wild emmer wheat [12, 13]. Other tetraploid wheat species have not been widely evaluated for reaction to FHB, although moderate or high levels of resistance of *T*. *carthlicum*, *T*. *polonicum*, and *T*. *turanicum* have been identified [7, 14]. Moreover, Wiwart *et al*. analyzed the morphometric parameters and chemical properties of grain of nine Polish wheat accessions in terms of the FHB tolerance [6]. The studied genotypes were relatively resistant to FHB and were considered as valuable gene source for breeding new wheat cultivars.

The very first step in species characterization is the karyotyping. Wheat karyotype analyses were began in the 1930s, when Sears isolated monosomics and telosomics [15]. Since the 1970s wheat chromosomes can be distinguished using the C-band or N-band technique [16, 17, 18].

Thereafter, molecular techniques were adapted into fluorescence *in situ* hybridization (FISH) method, which enabled direct localization of DNA sequences on chromosomes. Initially, probes, which were used in FISH experiments on plant chromosomes, contained conservative high-copy sequences, like 5S and 45S ribosomal RNA genes [19, 20]. What is more, the number and distribution of rDNA loci in the Triticeae species is reported to be a conservative feature of genomes or groups of homeologous chromosomes. These probes are often used as markers in phylogenetic studies, in the analysis of intra- and interspecific divergence, and in the elucidation of the origin of poliploid species [21, 22, 23, 24, 25]. Furthermore, some repetitive sequences such as pSc119.2 and pAs1 sequences [26] enabled to distinguish most of chromosome pairs of wheat. What is more, Cuadrado *et al*. [27, 28] used synthetic oligonucleotides (three base-pair repeats) to detect FISH signals on wheat chromosomes and found that the GAA microsatellite sequence is valuable to identify chromosomes belonging to the wheat A and B genomes. The (GAA)n FISH probe, widely used for the identification of different *Triticum* chromosomes [27, 28, 29, 30] gives strong pericentromeric and intercalary signals on all of the B chromosomes of hexaploid [27] and tetraploid [30] wheat. On the other hand, FISH techniques are widely used to conduct studies to screen various genomic libraries for useful clones [31]. Subclonings of BACs were made to identify the specific sequences that contributed to the FISH signals patterns on the wheat chromosomes [32]. The most discriminatory probes were generated by labelling pTa-535, pTa-713, and pTa-86 (homolog of pSc119.2 sequence) clones. Among them, the probe pTa-535 produced the highest number of signals on the wheat chromosomes. This clone is a 342 bp tandemly repeated DNA sequence, showing approximately 80% homology with clone pTa-173, a member of the *Afa*-family [32].

The genetic constitution among tetraploid *Triticum* species was established by domestication, selection bottlenecks, migrations and extensive gene flow [33]. Therefore, any useful data accrued in tetraploid wheats cytogenetics is likely to be desirable also to bread wheat breeders. Hence, the major purpose of this study was to trace the possible chromosome changes over the course of evolution, which could trigger intraspecific polymorphism in the organization of chromosomes of twenty different *T*. *polonicum* accessions using repetitive sequences and to compare the produced patterns to the chromosomes of other tetraploid wheats with the aim of accurate chromosome identification.

## Materials and Methods

### Plant material

**Fig 1:**
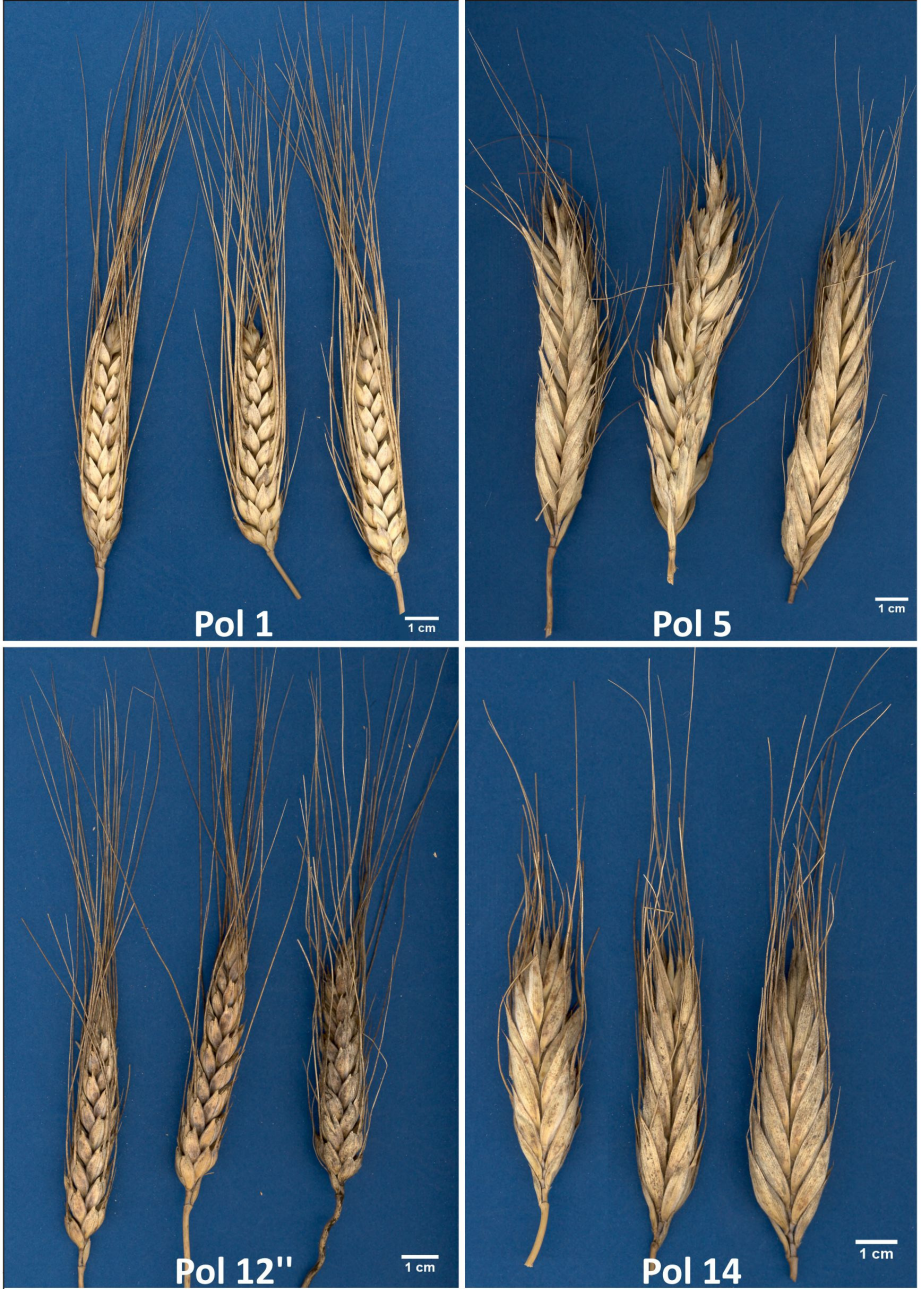
Spike morphology of selected *Trtitcum polonicum* accessions.

Plant material comprised twenty accessions of *T*. *polonicum*, four varieties of *T*. *durum* and one accession of *T*. *dicoccon* and *T*. *turanicum* (Table 1, Fig 1). Initially, plants of two *T*. *polonicum* accessions (Cltr 13919 and PI 167622) were diversified considering spike morphology. Hence, after several self-pollinations, we have selected three morphotypes from each of these accessions (Pol-12, Pol-12’, Pol-12” and Pol-14, Pol-14’, Pol-14”). The grain of *T*. *polonicum* and *T*. *durum* derived from field experiment established in the Experimental Station Bałcyny (53°36’N; 19°51’E) (Poland) whereas the grain of other species were grown in greenhouse at the Institute of Plant Genetic of the Polish Academy of Sciences.

**Table 1:** Origin description of the accessions used in this study.

	Genotype	Form	Accession number	Origin	Genebank
1	Pol-1	*Triticum polonicum* var. *novissimum* 'Litauischer'	PL21801	unknown	NCPGR/Radzików/Poland
2	Pol-2	*Triticum polonicum* var. *chrysospermum* 'Weihenstephanert'	PL 21802	unknown	NCPGR/Radzików/Poland
3	Pol-3	*Triticum polonicum* var. *chrysospermum* 'Dometzkoer'	PL 22488	unknown	NCPGR/Radzików/Poland
4	Pol-4	*Triticum polonicum* var. *polonicum* 'Passau'	PL 22479	unknown	NCPGR/Radzików/Poland
5	Pol-5	*Triticum polonicum* var. *chrysospermum* 'Schliephakes Riesenpolonicum'‘	PL 23047	unknown	NCPGR/Radzików/Poland
6	Pol-6	*Triticum polonicum* var. *heidelbergii*	PL 20770	unknown	NCPGR/Radzików/Poland
7	Pol-7	*Triticum polonicum* var. *chrysospermum* 'Mansholts White Sotare 1'	PL 22991	unknown	NCPGR/Radzików/Poland
8	Pol-8	*Triticum polonicum* var. *polonicum*	PL 22746	unknown	NCPGR/Radzików/Poland
9	Pol-9	*Triticum polonicum* Z -127 1	PL 22195	unknown	NCPGR/Radzików/Poland
10	Pol-11	*Triticum polonicum* ‘Milagre’	PI 56262	Portugal	NPGS/USA
11	Pol-12	*Triticum polonicum* 7498	Cltr 13919	Ethiopia	NPGS/USA
12	Pol-12’	*Triticum polonicum* 7498	Cltr 13919	Ethiopia	NPGS/USA
13	Pol-12”	*Triticum polonicum* 7498	Cltr 13919	Ethiopia	NPGS/USA
14	Pol-14	*Triticum polonicum* ‘Mika’	PI 167622	Turkey	NPGS/USA
15	Pol-14’	*Triticum polonicum* ‘Mika’	PI 167622	Turkey	NPGS/USA
16	Pol-14”	*Triticum polonicum* ‘Mika’	PI 167622	Turkey	NPGS/USA
17	Pol-16	*Triticum polonicum* ‘Arrancada’	PI 191881	Portugal	NPGS/USA
18	Pol-19	*Triticum polonicum* 149	PI 225335	Iran	NPGS/USA
19	Pol-22	*Triticum polonicum* 1-1-3490	PI 272570	Hungary	NPGS/USA
20	Pol-25	*Triticum polonicum* 42–1	PI 384265	Ethiopia	NPGS/USA
21	Dic-1	*Triticum dicoccon*	PI341805	Georgia	NPGS/USA
22	CS	*Triticum aestivum* subsp. *aestivum* ‘Chinese Spring’	Cltr 14108	USA	NPGS/USA
23	Dur-1	*Triticum durum* ‘Duromax’	n/a	Poland	University of Warmia and Mazury
24	Dur-2	*Triticum durum* ‘Duroflavus’	n/a	Poland	University of Warmia and Mazury
25	Dur-3	*Triticum durum* ‘Floradur’	n/a	Poland	University of Warmia and Mazury
26	Dur-4	*Triticum durum* ‘Malvadur’	n/a	Poland	University of Warmia and Mazury
27	Kam-1	*Triticum turanicum* ‘Kamut’	n/a	USA	University of Warmia and Mazury

### Chromosome preparation

Germination, metaphase accumulation and fixation procedures were carried out according to Kwiatek *et al*. [34]. Mitotic chromosome preparations were made from root tips digested in a mixture of enzymes, diluted with 0.01 M sodium citric buffer, containing 20% (v/v) pectinase (Sigma), 1% (w/v) cellulose (Calbiochem) and 1% (w/v) cellulase ‘Onozuka R-10’ (Serva). Chromosome preparation were made according to protocol reported by Heckmann *et al*. [35] with modifications made by Hesse (IPK Gatersleben, Germany, personal comm.). Root tips were placed on a slide in a drop of ice-cold 60% acetic acid and dispersed with a metal needle. Ice-cold acetic acid (60%) was added to the cell suspension, mixed and kept for 2 min at room temperature. Further, ice-cold 60% acetic acid was added and the slide was placed on a heating table (Medax) for 2 min. at 48°C. The slide was removed from the hot plate and 200μl ice-cold ethanol-acetic acid (3:1, v/v) was added to wash the slides. The slide was placed in 60% acetic acid for 8 minutes followed by briefly washing in 96% ethanol and then air dried.

### DNA probes

Six repetitive sequences: pTa-86, pTa-k374 (28S rDNA), pTa-465, pTa-535, pTa-k566 and pTa-713 were amplified from the clones originated from BAC library of wheat published by Komuro *et al*. [32] (http://www.ncbi.nlm.nih.gov/genbank). Sequences were amplified using primers (Table 2) designed with use of Primer3 [36] and their properties were tested using OligoCalc [37]. The PCR mix contained 0.75U of Taq DNA polymerase (ThermoScientific), 2.5 μl of PCR buffer, 12.5 pmol of forward/reverse primers (Table 2), 2.5 mMol each dNTP, and about 50 ng of wheat (cv. Chinese Spring) total genomic DNA. PCR conditions (SensoQuest Biomedizinische Elektronik, Goettingen, Germany) for all primer sets were optimized in initial studies (Table 2). The PCR reactions were made using program of: 95°C for 5 minutes, 35 cycles of 95°C for 30 seconds, annealing temperature appropriate for each primer pair (Table 2) for 30 seconds, 72°C for 90 seconds, and 72°C for 5 minutes. Amplification products were electrophoresed at 5 V/cm for about 2 hours in 2% agarose gel (Sigma), stained with Midori Green Direct DNA Stain (Nippon Genetic Europe GmbH), visualized under UV light and photographed. All sequences were labelled using nick translation kits according to manufacturer instructions. Probes pTa-86 and pTa-713 were labelled using digoxigenin-11-dUTP (Roche), whereas pTa-535 and pTa-k566 were labelled using tetramethyl-5-dUTP-rhodamine (Roche) and pTa-k374 and pTa-465 were labelled by Atto647 (Jena BioScience).

**Table 2:** Sequences of primers and PCR conditions for wheat repetitive sequences amplification.

Clone	NCBI Genbank sequence number	Primer sequences (5’ to 3’)	Annealing temperature (°C)	Product length (bp)
**pTa-86**	KC290896	ACGATTGACCAATCTCGGGG	58.5	531
		ACCGACCCAAATTACGAGAGT		
**pTa-k374**	KC290907	TTTCAACCAAGCGCGATGAC	59	690
		ATCAGCGGGGAAAGAAGACC		
**pTa-465**	KC290905	CCCACGCGTCTGTTTCC	59	527
		GTGGTCAAACGCTACACGG		
**pTa-535**	KC290894	GCATAGCATGTGCGAAAGAG	59	101
		TCGTCCGAAACCCTGATAC		
**pTa-k566**	KC290904	CTTTAGTCCACTACTCACC	51	251
		TTTCAAACACTTAGGCGA		
**pTa-713**	KC290900	GGGGCGGACGTCGTTG	59	337
		CCGTAAGATAGACAGGGTGGG		

### Fluorescence in situ hybridization

FISH procedure was similar to what was previously reported [38] with minor modifications. The hybridization mixture (40 μL per slide) contained 90 ng of each probe in the presence of salmon sperm DNA, 50% formamide, 2 × SSC, 10% dextran sulphate, and was denatured at 70°C for 10 minutes followed by incubation on ice for 5 minutes. Chromosomal DNA was denatured in the presence of the hybridization mixture at 75°C for 4 minutes and allowed to hybridize for 16 hours at 37°C. The post-hybridization washes were performed, according to Heslop-Harrison [39] at 42°C in 0,1 × SSC solution, providing the 73% stringency. Digoxigenin-11-dUTP detection was made using anti-digoxigenin-fluorescein antibody (Roche). Slides were examined with an Olympus XM10 CCD camera attached to an Olympus BX 61 automatic epifluorescence microscope. Image processing was carried out using Olympus Cell-F (version 3.1; Olympus Soft Imaging Solutions GmbH: Münster, Germany) imaging software and PaintShop Pro X5 software (version 15.0.0.183; Corel Corporation, Ottawa, Canada). After documentation of the FISH sites, the slides were washed (2 × 60 min in 4 × SSC Tween, 2 × 5 min in 2 × SSC, at room temperature) and after alcohol washes were dried and used for second FISH experiment. The order of FISH signal detections used was as follows. In the first FISH trial, pTa-86 (digoxigenin-11-dUTP), pTa-k374 (28S rDNA; Atto647) and pTa-535 (tetramethyl-5-dUTP-rhodamine) probes were applied. After removal of probes, pTa-465 (Atto647), pTa-k566 (tetramethyl-5-dUTP-rhodamine) and pTa-713 (digoxigenin-11-dUTP) probes were applied to the very same slides. The identification of particular chromosomes were made by comparing the signal pattern of probes hybridized to hexaploid wheat according to Komuro *et al*. [32] and Wiśniewska *et al*. [40].

## Results

The FISH experiment was carried out on 20 accessions of *Triticum polonicum*, 4 varieties of *T*. *durum* (Duroflavus, Duromax, Floradur and Malvadur), one accession of *T*. *dicoccon* and one variety of *T*. *turgidum* (Kamut) in order to detect chromosome polymorphisms. Three root meristems were collected from 10 plants of each accession. Slides were prepared from each of three root meristems and 10 metaphases per slide were analyzed and documented. To develop an informative combination of FISH markers that allows precise identification of all chromosomes, the following two sets of probes were tested: pTa-86 + pTa-k374 + pTa-535 and pTa-465 + pTa-566 + pTa-713. The identification of particular chromosomes were made by comparing the signal patterns according to Komuro et al. [32].

### A-genome chromosomes

**Fig 2:**
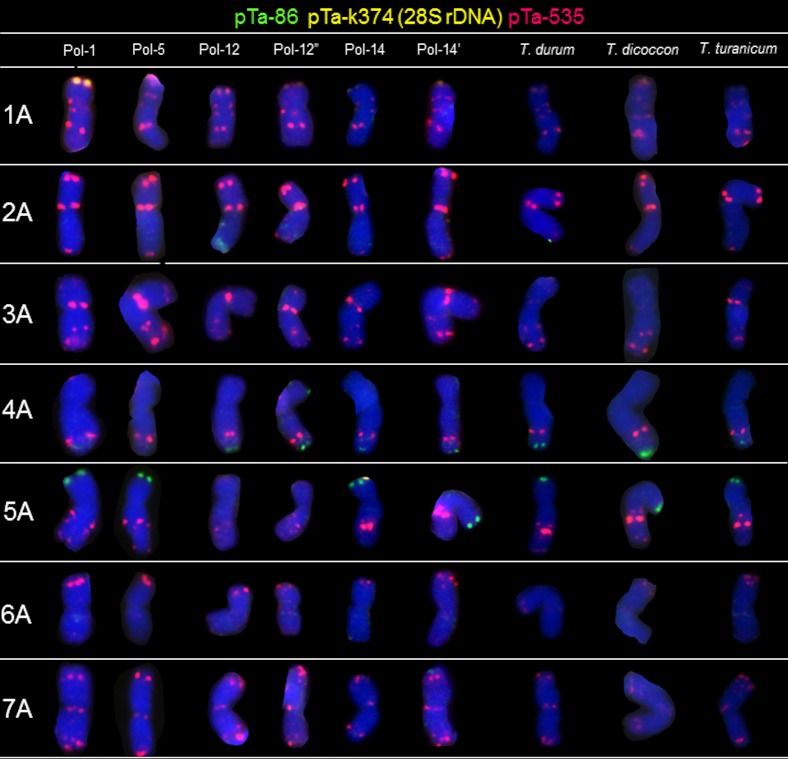
Karyograms of Pol-1, Pol-5, Pol-12, Pol-12”, Pol-14, Pol-14’, *T*. *durum*, *T*. *dicoccon* and *T*. *turanicum* showing A-genome chromosomes after FISH with pTa-86 (green), pTa-k374 (yellow) and pTa-535 (red) probes.

**Fig 3:**
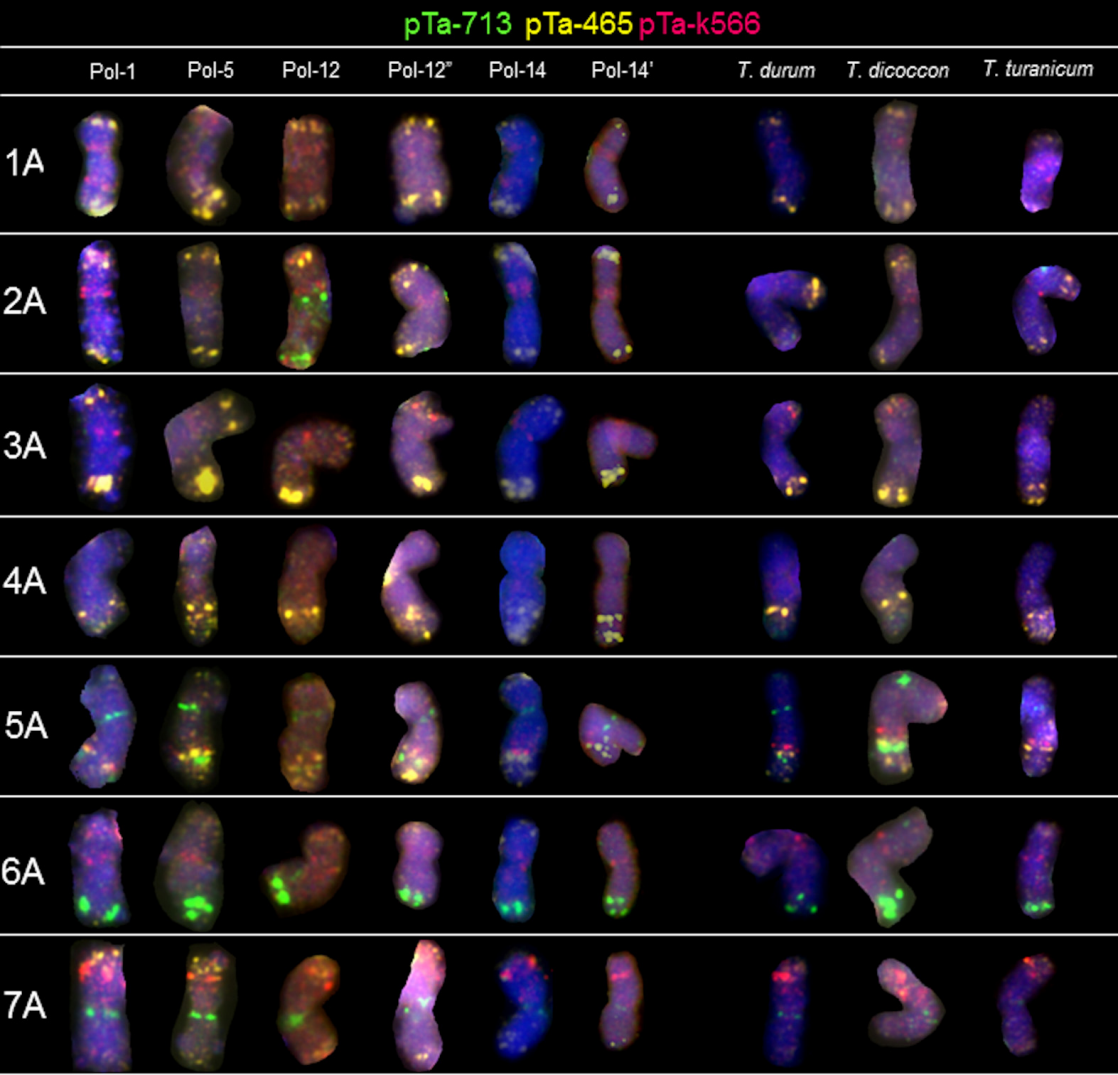
Karyograms of Pol-1, Pol-5, Pol-12, Pol-12”, Pol-14, Pol-14’, *T*. *durum*, *T*. *dicoccon* and *T*. *turanicum* showing A-genome chromosomes after second round of FISH with pTa-465 (yellow), pTa-k566 (red) and pTa-713 (green) probes.

Most of the Polish wheat chromosomes showed similar hybridization patterns compared to other tetraploid wheats, but some differences were observed among accessions of different origin and species. The pTa-k374 (28S rDNA) signal was observed in chromosome 1A only in Pol-1 (Fig 2). Only a few sites of the pTa-86 probe was identified in A-genome chromosomes. These signals were observed in the distal part of long arm of chromosome 4A of all accessions apart from the Pol-1 and Pol-5. What is more, this probe hybridized to the telomeric region region of short arm of chromosome 5A of all accessions, excluding Pol-12 and Pol-12’ (Fig 2). Probe pTa-86 pattern was also different concerning long arm of chromosome 2A of Pol-12 (Fig 2). Patterns of pTa-535 probe were more abundant in A-genome chromosomes. The most characteristic was 1A chromosome with pTa-535 sites located in pericentromeric and distal regions of both arms. The hybridization patterns were similar in all accessions, however in chromosome 5A the size of the site located in the middle of the long arm was variable. Moreover, no pTa-535 signals were observed in the pericentomeric and telomeric region of 3A chromosome of *T*. *durum* and *T*. *dicoccon*. FISH with pTa-465 resulted in clear, distinct signals, which were located mainly in the distal regions of chromosomes (Fig 3). The strongest signals appeared in the short arm of 2A and in the long arm of 3A chromosomes. The weaker signals were detected in the telomeres of all chromosomes. Moreover, the size of pTa-465 signals located in long arm of 5A and 7A chromosomes varied among different accessions. Sites of pTa-k566 probe were present in several chromosomes of Polish wheat. The most distinct signals were observed in the distal region of short arms of 7A, 3B and 4B chromosomes. Weak signals (Fig 2) are probably remnants of pTa-535 signals detected in the first round of FISH experiment. The most descriptive signal appeared in 7A chromosomes, which hybridized predominantly to the subtelomeric regions of short arms. Moreover, the pTa-k566 signals appeared in the short arm of 3A chromosomes of Pol-1, Pol-2, Pol-12, Pol-12’, Pol-12” and *T*. *durum*. The hybridization pattern with pTa-713 probe appeared in 5A, 6A and 7A chromosomes. It was similar in all genotypes of *T*. *polonicum* and other tetraploids. What is more, two additional pTa-713 signals were observed in the pericentromeric and telomeric regions of 2A chromosome of Pol-12 genotype.

### B-genome chromosomes

**Fig 4:**
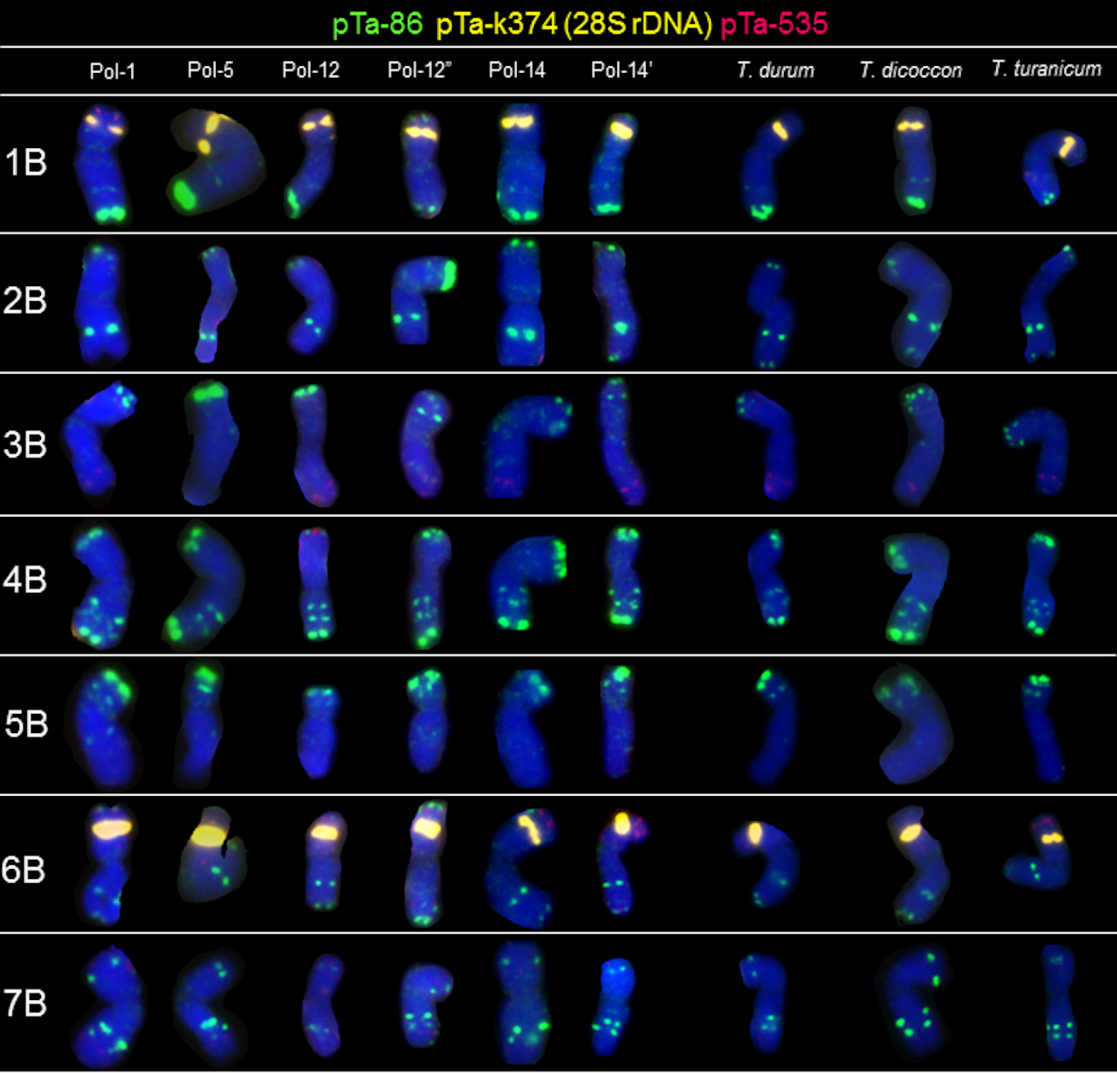
Karyograms of Pol-1, Pol-5, Pol-12, Pol-12”, Pol-14, Pol-14’, *T*. *durum*, *T*. *dicoccon* and *T*. *turanicum* showing B-genome chromosomes after FISH with pTa-86 (green), pTa-k374 (yellow) and pTa-535 (red) probes.

**Fig 5:**
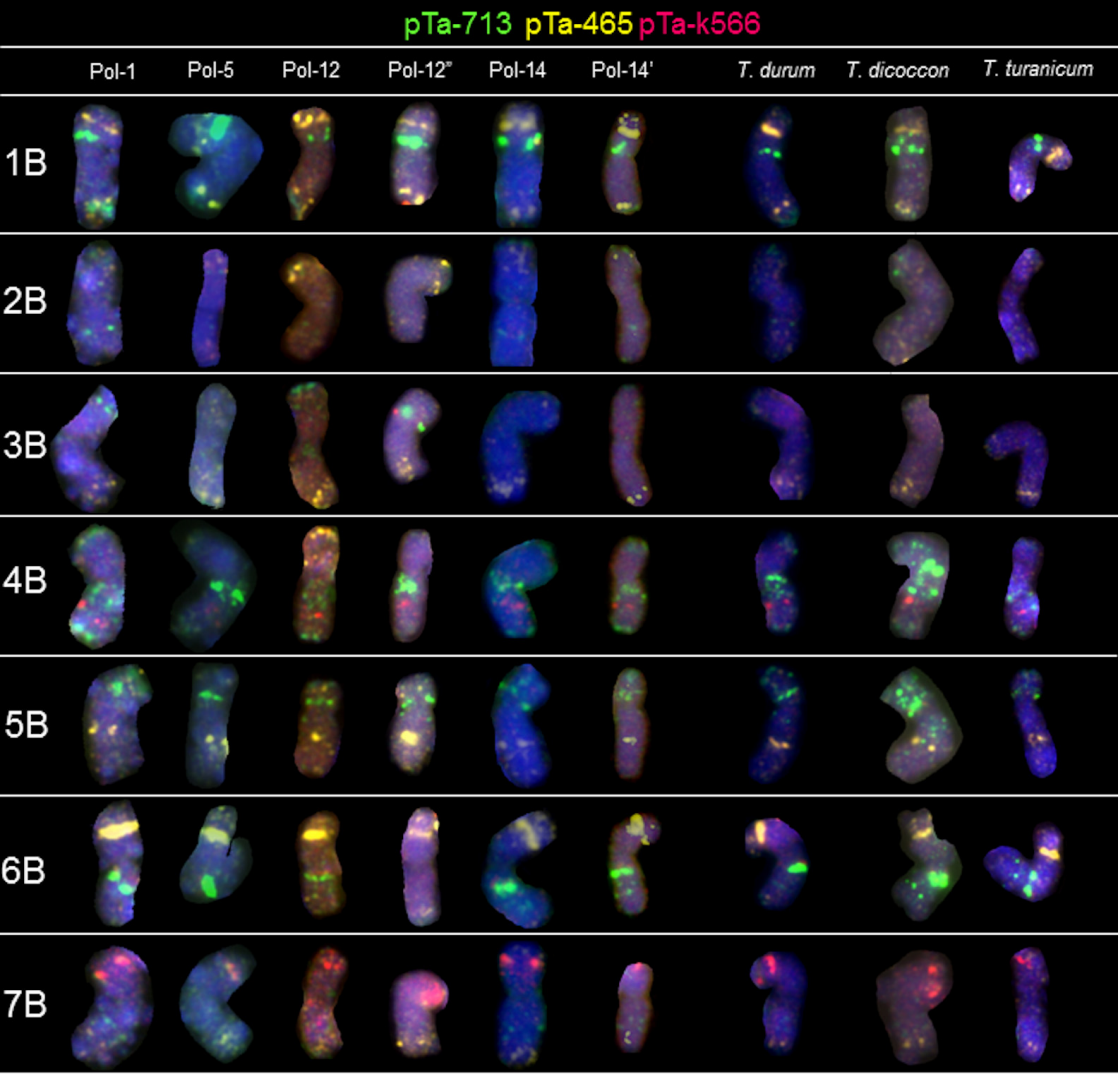
Karyograms of Pol-1, Pol-5, Pol-12, Pol-12”, Pol-14, Pol-14’, *T*. *durum*, *T*. *dicoccon* and *T*. *turanicum* showing B-genome chromosomes after second round of FISH with pTa-465 (yellow), pTa-k566 (red) and pTa-713 (green) probes.

Probe pTa-k374 (28S rDNA) generated the most conspicuous and characteristic signals in 1B and 6B chromosomes of all of 20 Polish wheat accessions, as well as in *T*. *durum*, *T*. *dococcon* and *T*. *turanicum* (Fig 4). The hybridization patterns of pTa-86 probe were more abundant in the B-genome chromosomes. The most characteristic, triple signals were observed in the arm ends of chromosome 4BL. This pattern appeared in 4B chromosomes of all accessions. Considering 5B chromosomes, the differences of pTa-86 probe site location appeared in the interstitial region of long arm. Hybridization of Polish wheat and other tetraploid wheats B-genome chromosomes with pTa-535 probe generated signals which were less abundant, when compared to A-genome chromosomes. Sites of pTa-535 were predominantly located in the telomeric regions of 3BL and 6BS chromosomes. However, in Pol-1 accession the pTa-86 and pTa-535 signals were exchanged considering telomeric regions of 1BS and 6BS chromosomes. What is more, in telomeric regions of 1BS and 6BS chromosomes of Pol-12” both sites of pTa-86 and pTa-535 are co-localized. From the other hand, additional pTa-535 signal was observed in telomeric region of 4BS of Pol-12 genotype (Fig 4). After reprobing, chromosomes of all accessions carried some remnants of NORs and pTa-86 telomeric sites, detected in the first round of hybridization (Fig 5). However, probe pTa-465 generated clear signals in the middle of long arm of 5B chromosomes and in the telomeric region of long arm of 7B chromosomes. Intraspecific polymorphism was detected considering the pTa-465 location in 2B chromosomes. This site was observed in the telomeric of short arm of 2B chromosomes of Pol-12, Pol-12’ and Pol-12”. What is more, weak and dispersed signals of this probe was also detected in distal regions of both arms of 2B chromosomes of Pol-5 and *T*. *dicoccon*. Hybridization of the pTa-k566 probe revealed a strong signals in the middle of long arm of 4B chromosomes and in the telomeric region of the short arm of 7B chromosomes of all accessions. However, pTa-k566 pattern was polymorphic considering short arms of 7B chromosomes of Pol-12 (Fig 5). Hybridization with probe pTa-713 generated large signals in the pericentromeric region on the short arm of 1B and 5B chromosomes, in the pericentomeric region of both arms of 4B chromosomes and in the middle of the long arm of the 6B chromosome (with the exception of Pol-12”). Intraspecific polymorphism was detected considering the pTa-713 location in 3B chromosomes (Fig 5).

## Discussion

In *Triticum* species, approximately 80% of the genome consists of repeated DNA sequences of varying degrees of reiteration and length [41]. The FISH procedure used in the present study can effectively map repetitive DNA sequences, which provides chromosomes of *Triticum* species to be recognized and classified [31]. Komuro *et al*. proposed a specific combination of pTa-535 + pTa-713 + pTa-86 probes to distinguish all wheat A-, B- and D-genome chromosomes [32]. The main goal of this study was to examine the differences and similarities in chromosomes organization among tetraploid wheats, which are believed as a potential source of genes that could be used in wheat breeding. For a comprehensive and detailed study we used six repetitive sequences: pTa-86, pTa-k374, pTa-465, pTa-535, pTa-k566 and pTa-713.

At first, we have taken a look on rDNA bearing chromosomes. In this purpose, we have used probe pTa-k374, which is a 28S rDNA sequence and map NOR loci in wheat [32]. The number of pTa-k374 probe signals did not differ between Polish wheat accessions and other tetraploid wheats. Predominantly, we have observed the 28S rDNA signals only on 1B and 6B chromosomes with one exception of *T*. *polonicum* ‘Pol-1’, where a minor signal on 1A chromosomes were detected. The A-genome of polyploid wheats originated from *T*. *urartu* [24, 25], where two pairs of 28S rDNA signals are located on 1A^u^ and 5A^u^ chromosomes. The intensity of 28S rDNA signals of A-genome chromosomes of *T*. *urartu* are usually equal [42, 43]. However, it could differ between 1A and 5A chromosomes of *T*. *urartu*. Badaeva *et al*. [25] detected a significant size reduction of 28SrDNA signals on one of the NOR-bearing chromosomes of *T*. *urartu* (PI 428311). In our study of tetraploid wheats we have not observed any signals in 1A and 5A chromosomes, except 1A chromosome of Pol-1 genotype carrying pTa-374 (28S rDNA) site. The phenomenon of rDNA locus loss in polyploid species is quite common [44, 45] and may be related to asymmetric transcription and epigenetic modifications caused by the polyploid formation. Guo *et al*. [46] observed A-genome and D-genome sets of parental nucleolus organizing regions (NORs), which were expressed after hybridization, but asymmetric silencing of one parental NOR was immediately induced by chromosome doubling, and reversing the ploidy status could not reactivate silenced NORs. The authors indicated, that asymmetric epigenetic modification and elimination of rDNA sequences between different donor genomes may lead to stable allopolyploid wheat with increased differentiation and diversity.

The next step of our study was to apply FISH markers for precise comparison of chromosomes of tetraploid wheats. The most distinctive signals on multiple chromosome arms were revealed using pTa-86 (pSc119.2 related sequence), pTa-535 (342-bp tandem repeat) and pTa-713 (71-bp tandem repeat) [32]. This probes enabled to capture the differences among different *T*. *polonicum* accessions, especially in 2A, 4A, 5A, 6A, 2B, 4B, 6B and 7B chromosomes. The signal patterns of pTa-86 and pTa-535 probes revealed probable evolutionary exchange of the telomeric region between short arms of 1B and 6B chromosomes of Pol-1 (*Triticum polonicum* var. *novissimum* 'Litauischer'). What is more, our analysis indicate that telomeric region of the short arm of 6B chromosome is polymorphic considering pTa-86, pTa-465, pTa-535 and pTa-713 signal locations. The FISH experiment with six probes revealed some polymorphisms in chomosome 5A of Pol-1, Pol-12, Pol-12’, Pol-12” and the rest of analyzed tetraploids. In contrast, the repetitive sequences distribution in 2B chromosomes distinguished Pol-1 genotype from Pol-12, Pol-12’, Pol-12” and Pol-5 genotypes. Basically, FISH analysis showed similarities in organization of particular chromosomes of certain *T*. *polonicum* genotypes compared to other tetraploid species. However, there are some polymorphisms in signal patterns such as: pTa-k566 FISH pattern of 3A chromosome of Pol-1 was different compared to *T*. *dicoccon*, *T*. *durum* and *T*. *turanicum*.

Considering all tetraploid accessions, it could be said that signal patterns were more diversified considering B-genome chromosomes. The biggest differences were observed in pTa-86 signal distribution in 2B, 3B, 5B and 7B chromosomes. This could be related with the B-genome diversity which much higher than A-genome genomic variability [47, 48, 49, 50]. According to Akhunov *et al*. [51], who studied the variation in diversity of SNP markers in individual chromosomes of bread wheat and emmer wheat, chromosome 2B had higher diversity and chromosome 4B had lower diversity than the rest of the chromosomes, which is in parallel with the analysis of repetitive sequences locations in chromosomes made in this study.

Taking into consideration the FISH patterns of pTa-465 and pTa-k566 signals, the hybridization patterns were usually similar among analysed accessions, with only a few polymorphisms detected (6A and 7B chromosomes). However, those probes can be considered as a supportive set of FISH markers, which confirm the chromosome identification. In example, a set of probes pTa-86, pTa-535 and pTa-713 reveals quite similar hybridization patterns in 2B and 7B chromosomes, but additional pattern of pTa-465 and pTa-k566 probe signals resolve the doubts. Moreover, hybridization with pTa-713 probe is helpful in correct identification of 1B and 6B chromosomes.

In conclusion, FISH with particular probe combinations exhibits similarities and differences in chromosome organization between tetraploid *Triticum* species. A set of pTa-86 + pTa-535 + pTa-713 probes were the best informative among 6 DNA probes tested. Additionally, chromosomes 1A, 1B and 6B can be identified by using pTa-k374 probe, which is similar to 28S rDNA sequence. The application of six probes enabled to detect signals that differs the 20 accessions of *T*. *polonicum* and, what is more, allowed to compare the chromosome organization in selected tetraploid species of *Triticum* genus. Furthermore, pTa-465 and pTa-k566 probes are helpful for the detection of similar organized chromosomes. The interest of food industry derives from the necessity to increase the diversification of commercialized products. This data can be important for breeders looks at genetic diversity present in the ancient wheats germplasm as an important reservoir of economically useful genes.
